# Supplementary material for: The interactome of intact mitochondria by cross-linking mass spectrometry provides evidence for coexisting respiratory supercomplexes
Source: Mol Cell Proteomics. 2017 Dec 8;17(2):216–32. doi: 10.1074/mcp.RA117.000470 (PMC5795388; doi:10.1074/mcp.RA117.000470)
Supplement: Supplemental Data [file supp_RA117.000470_133922_0_supp_23978_fzffwf.zip › spectra_annotation/mito_spectra_annotation/451-2-9-1-33-1.pdf]

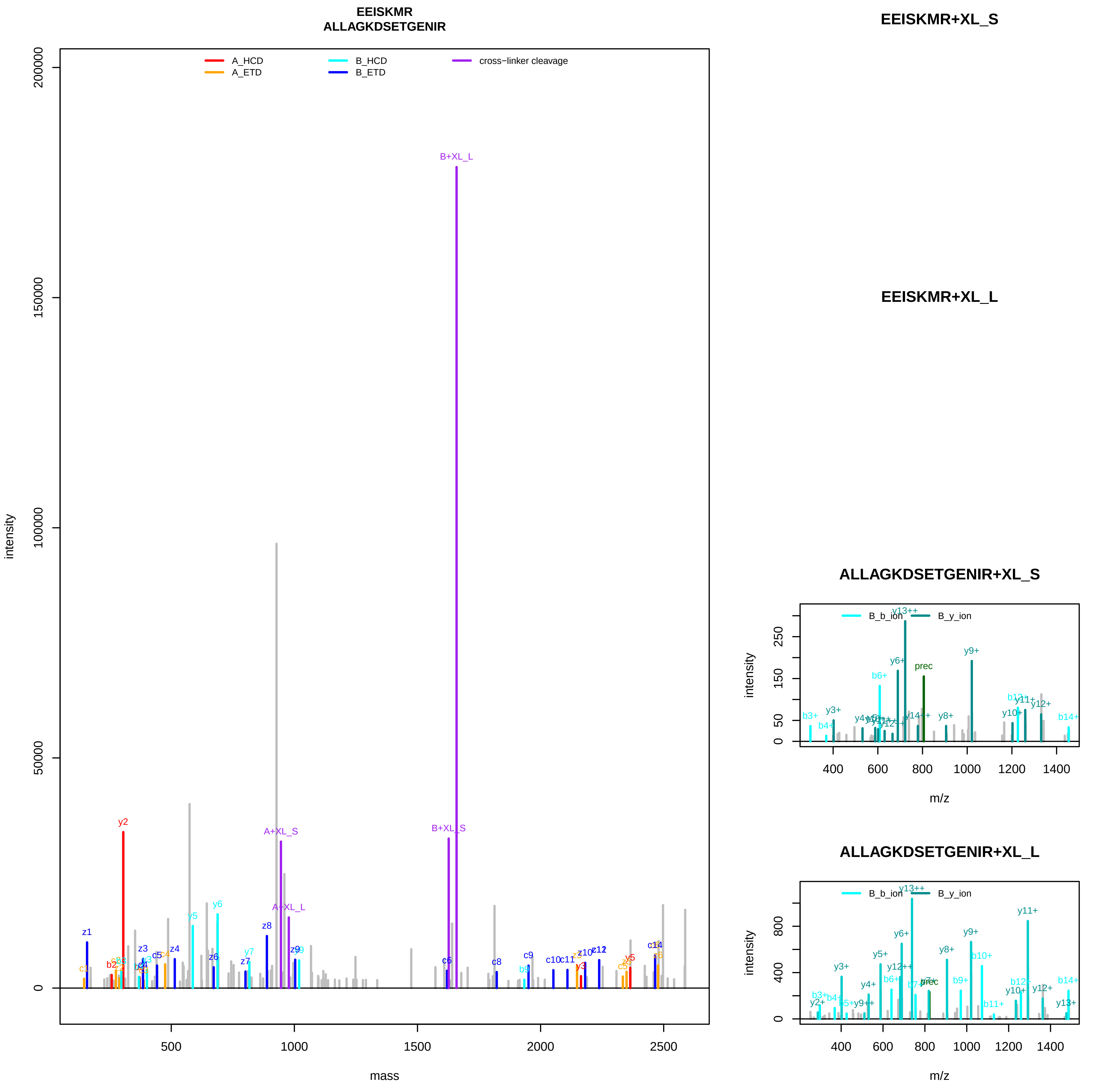
EEISKMR
ALLAGKDSETGENIR
A_HCD
A_ETD
B_HCD
B_ETD
cross−linker cleavage
B+XL_L
B+XL_S
A+XL_S
A+XL_L
y2
y5
y6
y7
z1
z3
z4
z6
z7
z8
z9
z10
z12
c4
c5
c6
c8
c9
c10
c11
c14
intensity
mass
0
50000
100000
150000
200000
500
1000
1500
2000
2500
EEISKMR+XL_S
EEISKMR+XL_L
ALLAGKDSETGENIR+XL_S
B_b_ion
B_y_ion
y13++
y9+
y6+
b6+
prec
y3+
y8+
b3+
b4+
b14+
y10+
y11+
y12+
m/z
0
50
150
250
400
600
800
1000
1200
1400
ALLAGKDSETGENIR+XL_L
B_b_ion
B_y_ion
y13++
y11+
y9+
y6+
y5+
y3+
y8+
b10+
y12++
b6+
y4+
b9+
b12+
b14+
y10+
y12+
b3+
b4+
b11+
y2+
y13+
m/z
0
400
800
400
600
800
1000
1200
1400
